# Spatial Promoter Recognition Signatures May Enhance Transcription Factor Specificity in Yeast

**DOI:** 10.1371/journal.pone.0053778

**Published:** 2013-01-08

**Authors:** Richard W. Lusk, Michael B. Eisen

**Affiliations:** 1 Department of Ecology & Evolutionary Biology, University of Michigan, Ann Arbor, Michigan, United States of America; 2 Department of Molecular & Cell Biology, University of California, Berkeley, California, United States of America; 3 Howard Hughes Medical Institute, University of California, Berkeley, California, United States of America; University of Leuven, Belgium

## Abstract

The short length and high degeneracy of sites recognized by DNA-binding transcription factors limit the amount of information they can carry, and individual sites are rarely sufficient to mediate the regulation of specific targets. Computational analysis of microbial genomes has suggested that many factors function optimally when in a particular orientation and position with respect to their target promoters. To investigate this further, we developed and trained spatial models of binding site positioning and applied them to the genome of the yeast *Saccharomyces cerevisiae*. We found evidence of non-random organization of sites within promoters, differences in binding site density, or both for thirty-eight transcription factors. We show that these signatures allow transcription factors with substantial differences in binding site specificity to share similar promoter specificities. We illustrate how spatial information dictating the positioning and density of binding sites can in principle increase the information available to the organism for differentiating a transcription factor’s true targets, and we indicate how this information could potentially be leveraged for the same purpose in bioinformatic analyses.

## Introduction

A typical transcription factor in the yeast *Saccharomyces cerevisiae* binds to short, six to ten base pair sequences in promoters [1], with the strength of this binding depending on the specific sequence of the site [2], [3]. Both strongly- and weakly-bound sites can impact the expression of adjacent genes [4], [5]. While this flexibility to bind different short sequences is part of what allows genes to be precisely regulated [5], it also makes potential binding sites quite common in the genome, raising the question of how, or whether, these short sequences alone are sufficiently informative for transcription factors to distinguish target from non-target promoters.

Wunderlich and Mirny examined this question formally within the framework of information theory [6]. Information theory is concerned with quantifying the information carried by codes such as DNA, and it has a rich history in the analysis of transcription factor binding sites [2], [7]–[9]. They found that binding sites in eukaryotes carry far less information than would be required to accurately differentiate them from the rest of the genome, suggesting that transcription factors must bind promiscuously to nonfunctional sites.

Nonfunctional binding appears to be pervasive in higher eukaryotes [10], but even in *S. cerevisiae*, with its relatively small genome, binding poorly predicts function. Hu et al. [11] found that binding of a transcription factor to a locus as measured by ChIP-chip typically does not predict that locus to exhibit a significant expression change upon deletion of that factor. Some of this discrepancy could be explained by noise in the ChIP dataset or by compensating effects in *cis* or *trans*. But even after mitigating these effects, Hu et al. found many examples of promoters that appeared to be bound by a transcription factor but not regulated by it. This suggests that functional targets carry additional contextual information beyond the set of bases in their binding sites that determine whether a given binding site affects regulation.

It is possible that, by treating positions within the binding site independently, we underestimate the information they carry. There is now considerable evidence that positions within binding sites do not affect binding independently [12]–[14]. However, the magnitude of this effect is small for most factors, and position-independent weight matrices appear to describe the bulk of variation in binding affinity [15]–[17].

Alternatively, the additional information required to explain factor specificity could be found outside individual binding sites. Several factors are known to interact with other factors and components of the transcriptional machinery in ways that affect how their location, orientation, and/or density impact their binding and effect on expression. For example, Rap1 activity was shown to be markedly different depending on which strand its sites were placed and whether or not they appeared as a tandem pair [18], [19]. Reb1 and Abf1 play critical roles in the creation and positioning of nucleosome free regions [20], which are precisely positioned with respect to the transcription start site [21]. This role suggests that, in turn, Reb1 and Abf1 binding sites must be precisely placed in order to function. Other proteins may be less precisely spaced: the homologous factors Met31 and Met32 bind DNA but have no intrinsic ability to activate transcription; their role is to recruit the co-activator Met4 to this sequence [22], and this indirect interaction may afford some flexibility in their positioning.

Finally, beginning with experiments using artificial constructs [23], cooperativity driven by binding site density has been thought to play a role in promoter recognition: if the relationship between site number and expression effect is nonlinear, then spurious single sites can be made inconsequential. Many transcription factors, such as Rap1 discussed above, have been shown to bind as dimers. Other factors, such as Rtg1 and, in *A. nidulans*, AlcR, bind as monomers but, notably, only affect expression in promoters with a sufficiently high number of binding sites [24]–[26]. Cooperative effects in these cases could be driven by less precise protein-protein interactions or indirectly, through competition with nucleosomes [27], [28]. Taken together, these characteristic requirements of positioning and/or density could create a promoter-recognition ‘signature’ for a factor that could render many non-target binding sites irrelevant and increase the discriminatory information available for recognizing true target promoters.

Relatively few transcription factors are understood to this level of mechanistic detail, but several computational works have suggested that these promoter recognition signatures could be a common property. Elemento et al. [29] used a mutual information approach to simultaneously discover expression-influencing consensus sequences and their location and strand biases, showing that, for a large fraction of the consensus sequences they uncovered, location and often strand informed expression. Following up this work in a large number of factors, Westholm et al. [30] found that the location and strand of many consensus sequences are distributed non-randomly within promoters. Erb and van Nimwegen incorporated weight matrices and evolutionary information into a similar analysis, allowing them to divide transcription factors into different classes based upon the positional biases of their binding sites that are suggestive of different mechanisms of regulation [31], [32].

Here we approach the problem from a different perspective, focusing on the properties of whole promoters rather than aggregate properties of individual binding sites. We develop a statistical model of promoter signatures for a wide variety of transcription factors in *S. cerevisiae*, integrating over strong and weak sites and describing factor-specific biases in site location, strand bias, and density. Using this model, we show that spatial information, in particular site density, appears to play a role in the function of the typical yeast transcription factor. Framing our spatial model in the context of information, we show that this spatial information can, in principle, allow transcription factors that weakly specify their individual binding sites to share the same promoter specificity as transcription factors with much more strongly specified binding sites. We illustrate the discriminatory utility of spatial information using expression changes in transcription factor deletion strains, showing that its target predictions are for most factors more strongly associated with expression change than are predictions from spatially naïve models.

## Results

### Description of the Model

**Figure 1:**
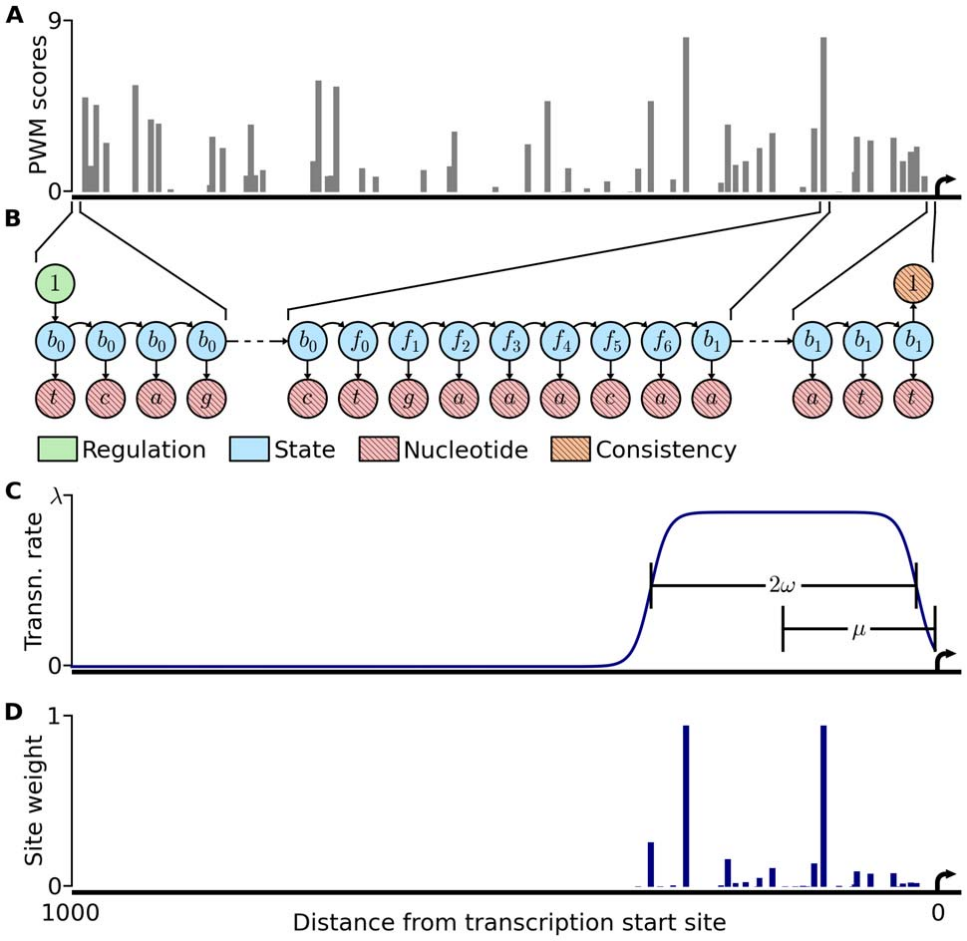
Description of the model. (A) One kilobase upstream of the transcription start site of YPL192C is depicted, with PWM-scores of putative Ste12 binding sites plotted in gray. The transcription start site is represented by an arrow. (B) A sample state configuration for the model is shown. Variables are represented as circles, with hatching added to variables considered to be ‘observed.’ As described in detail in Methods, the binary ‘regulation’ variable, in green, emits a series of ‘site’ variables (blue), each corresponding to and emitting a single nucleotide (red) in the promoter. The middle segment highlights how a background b_0_ state transitions to a series of frequency matrix states, which in turns transitions to a background b_1_ state. This b_1_ value is carried, as shown, to the end of the sequence, where it emits a final background nucleotide and the observed value of 1 for the ‘consistency’ state, in orange. This consistency state takes value one if the final state variable takes a value of either b_1_ or b_x_, ensuring that the original ‘regulation’ variable specifies whether or not a binding site is emitted. The frequency matrix states shown here correspond to the position of one of the two highest-scoring matches to the Ste12 motif; here they emit the consensus TGAAACA sequence observed on the forward strand of the YPL192C promoter. (C) The probability of transition from a background state to a frequency state depends on the position of the nucleotide. Here we depict the final spatial model for Ste12, highlighting how the fitted parameters μ and ω specify the center and the width of the spatial distribution of emitted binding sites. The maximum height of the plateau corresponds to the parameter λ, which determines the rate at which binding sites are emitted. Not shown are the parameters ρ, which determines the probability that any site at all will be emitted, τ, which determines the extent of the strand bias of emitted sites, and η, a free parameter that determines the slope of the curve up to the plateau. (D) The model incorporates position weight matrix information (depicted in 1A) and spatial information (depicted in 1C) to arrive at a weight for each putative binding site. Here we plot, for each position, the expected value that the state variable corresponds to the beginning of a binding site.

We use a hidden Markov model to describe the positions of binding sites for a single factor within a set of promoters (fig. 1). For each promoter, a single binary ‘regulation’ (R) state determines whether or not the emitted sequence will carry the factor’s promoter signature. A set of hidden ‘site’ (S) states generate the observed nucleotide (N) states, one per position in the promoter, according to either a background nucleotide distribution or the appropriate position-specific distribution found within the factor’s binding site. A ‘consistency’ (C) state generated by the last S state ensures that at least one binding site is emitted if a promoter is classified by the R state as being regulated by the factor (see Methods). We train five parameters: ρ, estimating the fraction of sequences in the training set that carry the factor’s signature, μ and ω, describing the center and width of a region enriched for the factor’s binding sites, τ, estimating these sites’ strand bias, and λ, a rate parameter which describes the density of sites in the enriched region. As this Poisson-like parameter cannot easily describe the plausible case in which a transcription factor relies on strictly one binding site for recognition, we also train a similar model which generates a single binding site per promoter. We also incorporate a free parameter η which determines the slope of the transition to the enriched region (fig. 1C, see Methods). We formally describe these models, as well as fitting and selection, in the methods section.

These models have several useful properties. They can take advantage of position weight matrices rather than consensus sequences, and while remaining computationally tractable, they are able to integrate over strong and weak binding sites. As the true shape of the spatial distribution of any given factor’s binding sites may differ between factors [32], [33], we chose to use a relatively flat distribution, creating a plateau-like region enriched for binding sites (figure 1C). While it is possible to specify a perfectly flat region with sharp sides, we found that a gentler transition to the enriched region aided parameter optimization considerably.

### Promoter Recognition Signatures are Common and Differ between Transcription Factors

We used the Harbison et al. [34] ChIP-chip binding data and position weight matrices from the MacIsaac et al. [35] analysis as the basis for our model training. We filtered the binding data in four ways. First, as the position of transcription factor binding sites is much more strongly related to the transcription start site than to the translation start site [36], we removed 5′ untranslated regions from our data. Second, we only used intergenic regions containing highly-conserved binding events [35] to help remove bound but functionally unimportant sites. Third, although we placed a conservative upper limit on the length of the promoter at 1,000 base pairs, ORFs and other annotated functional sequences were replaced by randomly generated background sequence. Finally, we removed divergently transcribed genes so that we could unambiguously describe the positioning of binding sites relative to a single ORF.

**Figure 2:**
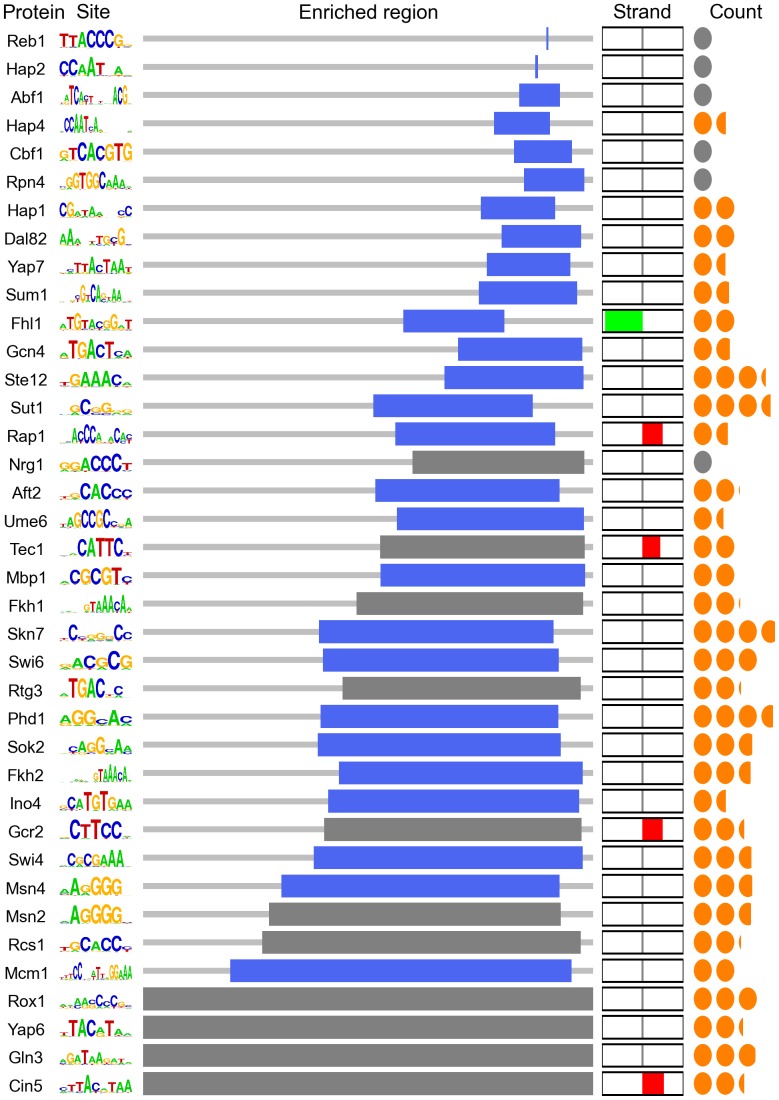
Description of promoter signatures. Promoter signatures for all transcription factors with more than twenty screened bound intergenic regions, excluding those with trainable signatures in unbound regions. Sequence logos depict the frequency matrices described in the main text. The blue region corresponds to the site-enriched plateau illustrated in figure 1C: it is centered at the location parameter μ and shows the range from μ−ω to μ+ω. If the region is gray, then either we were unable to find statistically significant support for training the parameters μ and ω (bottom four cases) or these trained parameters failed our shuffling test (top seven cases), indicating for these factors that promoter lengths alone are sufficient to explain their observed spatial restriction. The strand column depicts strand bias, from 100% reverse-strand bias (green) to 100% forward-strand bias (red). Circles in the count column depict the expected number of binding sites per promoter. Gray circles correspond to those sequences that better fit the monosite model, having strictly one site per promoter.

We fitted our model to all ChIP-chip sets having at least twenty promoters meeting our criteria (fig. 2). We confirm [30], [32], [33], [36], [37] the presence of factor-characteristic spatial biases of binding sites for a large number of transcription factors. For each factor, we used likelihood ratio tests over a series of nested models to determine the significance of parameters describing the factor’s strand and spacing preferences. As describing binding site density requires a slightly different model structure, we determined the utility of this parameter using an information criterion.

Although most factors displayed a nonrandom spatial distribution of binding sites, and there appears to be a diversity of such distributions, we wondered whether this diversity could arise as an artifact of differences in intergenic sequence length. For instance, the typical intergenic region bound by Rpn4 is substantially shorter than the typical intergenic region across the genome; even if Rpn4 sites were randomly scattered throughout this region, we would expect our model to find Rpn4 sites to be more spatially restricted than most other transcription factor binding sites. To control for this effect, we also trained our model on data sets with scrambled binding site positions but conserved promoter lengths and binding site number and strength. Parameterizing null models of site positioning with the location (μ) and width (ω) of the binding-site-enriched region learned from these scrambled data, we used likelihood ratio tests to show that values trained from the unscrambled data fit significantly better for all but seven transcription factors, suggesting that the spatial restrictions we report here are driven by more than intergenic sequence length.

We used unbound sequences to assess the impact of weakly or incorrectly specified matrices. If a transcription factor’s frequency matrix is likely to appear anywhere, perhaps due to a flaw in our representation of background sequence, then our model could associate with that matrix as well-populated but ultimately meaningless spatial signature. We compensated for this property by fitting our model, for each factor, to regions not bound by that factor in any tested condition. If we were able to discover any putative signature populated to an appreciable level in these data, we consider the original signature suspect and discard it (see Methods). Although this test is conservative, as we expect a substantial amount of condition-specific binding to have been missed by these ChIP-chip data, only a handful of factors’ spatial signatures failed this test (Dig1, Ndd1, Pho2, Yap5, Mot3, and Swi5).

**Figure 3:**
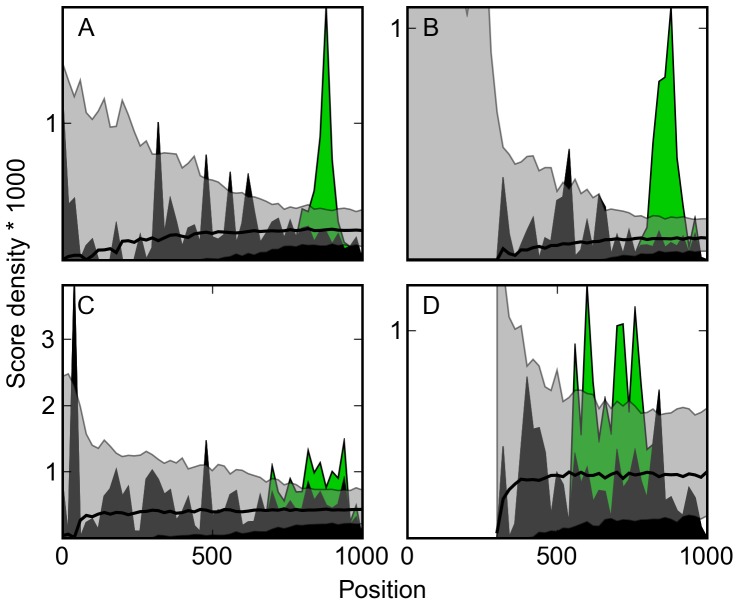
Transcription factors exhibit a diversity of spatial preferences. Score density is plotted against position. Score density is defined as the sum of positive log-two position weight matrix scores in a twenty base window, divided by the total number of possible binding site positions within that window of the training data. The black line is the simulated background score density; the gray area is the 95% confidence interval about that line. Confidence intervals are wide in windows far from the transcription start site due to the low number of intergenic regions in the training data reaching this distance. The green area is weighted by the model to be part of the promoter-signature distribution; the black area is weighted by the model to be part of the background distribution. Depicted factors are (a) Reb1, (b) Abf1, (c) Gcn4, and (d) Fhl1. No intergenic region used to train Fhl1’s spatial signature is as long as 1,000 base pairs, creating a blank area.

The tested set of factors exhibits a diversity of spatial patterns. Several factors have sites tightly positioned in relation to the transcription start site. Notably, we recover the hypothesized tight spatial constraint of Reb1 and Abf1 (fig. 3A, B). Several other factors, including Cbf1, Rpn4, and members of the Hap2/3/4/5 complex, also appeared to recognize their targets according to tight special constraints, and we hypothesize that they may operate under similar mechanistic pressure. Other factors, such as Gcn4, do so more broadly (fig. 3C). Most factors’ binding sites were found almost up to the start of transcription, but the site associated with Fhl1 (fig. 3D) was a notable exception, although questions have been raised about whether Fhl1 binds to the DNA directly [38]. While relatively few factors’ sites exhibited a significant strand bias, we recovered the characteristic bias of Rap1 sites.

Although analogous parameters describing site density can be found in enhancer prediction algorithms designed for higher eukaryotes (e.g. [39]–[43]), our model is to our knowledge the first description of location bias to explicitly account for binding site density in yeast promoters. While some factors, usually those that appear to be strongly spatially constrained, appear to recognize a single site within promoters, the typical factor appears to rely on multiple sites. If multiple sites are a functional necessity for a promoter’s recognition by a transcription factor, then we have, immediately, an intuitive means for increasing a transcription factor’s promoter specificity.

### Spatial Information can Offset Weak Binding Information

Many eukaryotic transcription factors have binding sites that are short enough, and nonspecific enough, that identical copies of functional sites often appear in non-target promoters and enhancers. Examining this formally using information theory, Wunderlich and Mirny [6] demonstrated that, unlike those in prokaryotes, virtually all transcription factors in yeast and other eukaryotes do not contain enough information to differentiate their targets from background sequence on the basis of their individual binding sites. This suggests that these factors must take advantage of other, additional information to prevent widespread misregulation of nontarget promoters.

The information content of a transcription factor’s binding sites can be quantified as the Kullback-Liebler (KL) divergence between the distribution of bases found in these sites and the distribution of those bases within the genome [2]. This information content has been used as a metric to compare the specificity of different transcription factors and forms the theoretical basis of sequence logos and position weight matrices, the most common representations of transcription factor binding sites.

We desired to use this framework to compare the specificity of our predicted sequence signatures of target recognition and quantify the increase in specificity they could potentially provide over binding sites alone. To this end, we developed a means to calculate the KL divergence between each predicted sequence signature and a background distribution of completely random promoter sequences, providing us with a means to quantify the information provided by our sequence signatures. For comparison, we created and repeated this calculation for artificial density- and spacing-agnostic signatures containing a single binding site in each promoter: by comparing, for each factor, the information contained in the full model with the spacing-agnostic model, we learn how much information is provided by each spatial signature. We note that while exactly calculating the true value of these metrics is all but impossible, as it requires integrating over all possible promoter sequences, a sampling approach enabled us to calculate approximate values (see Methods).

**Figure 4:**
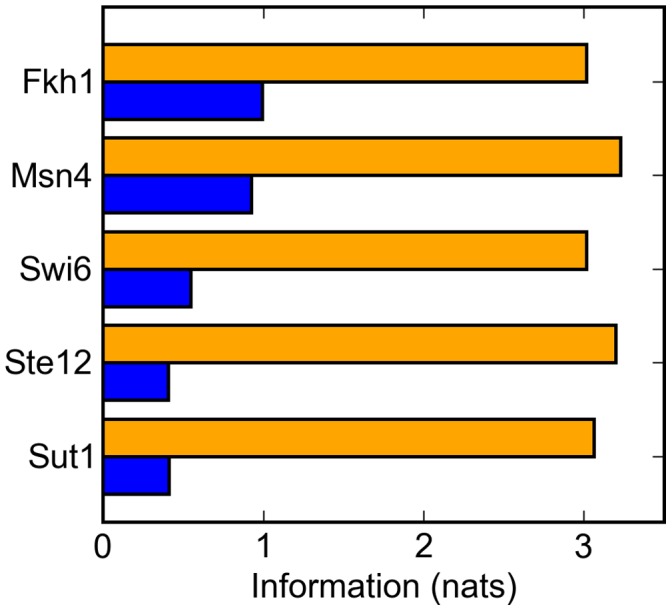
Promoter signatures compensate for and increase the information available to weakly specified binding sites. The specificity of a transcription factor is measured by the Kullback-Leibler divergence between the distribution of possible binding sites and a distribution of background sequences. Here we have calculated an analogous measure to quantify the specificity of our of different spatial signature models: each model emits a distribution of possible promoter sequences, allowing us to approximate the Kullback-Leibler divergence, and hence the specificity, separating this promoter sequence distribution from a distribution of background sequences. This measure of information is plotted in orange here for each of five factors. For comparison, in blue, we plot the information carried by a simpler model. This model emits sequences with only a single site without strand bias or spatial restriction, and as such the information of the promoter distribution is solely the product of the information carried by the binding site. In every case, spacing, strand, and/or density substantially increases the information carried by the model. For these five factors, these restrictions allow them to share essentially the same promoter-level information content despite their diversity of binding site specificities.

As individual binding sites form the building blocks of any spatial model, we expect that a signature’s specificity is driven in large part by the specificity of its component sites. Indeed, we observed a strong correlation between the information content of our sequence signatures and the information contents of their respective sequence motifs (r^2^ = .67, p = 6.51 * 10^−12^, figure S1): factors that have well-specified binding sites tend, on average, to also have well-specified promoter signatures. Even so, there exists considerable variation in matrix specificity given a certain promoter recognition specificity. In figure 4, we illustrate five factors that, while their signatures share approximately the same discriminatory information, have substantially different abilities to discriminate between promoters when spacing, strand, and site density are disregarded. Restriction of these properties is thus in principle able to compensate for a weakly specified frequency matrix.

We also note that, for these factors and nearly all others, overall specificity is greatly increased by the addition of promoter recognition signatures. While factors that rely on one site, without a strand bias, such as Rpn4, gain only a modest specificity increase due to their spatial restriction, most appear to rely on several and show an accordingly large increase in specificity. For instance, Msn4’s binding site alone carries approximately one nat of information, which, in theory, is only sufficient to differentiate roughly one third of the genome as its targets–a far larger role than Msn4, or any other sequence-specific transcription factor in yeast, is expected to play. However, its promoter recognition signature carries more than three nats of information, thought to be sufficient to differentiate roughly 250 targets, only slightly larger than the approximately 200 true targets Msn4 is expected to have [44]–[46]. For reasons we elaborate upon in the Discussion, we do not expect most factors to share this match between calculated specificity and true target size. Nevertheless, these promoter signature driven increases in specificity illustrate a route by which transcription factors can identify their targets and, as we show in the next section, could be helpful for bioinformaticians interested in doing the same.

### Promoter Recognition Signatures Predict Expression Change in Factor Deletion Mutants

To test whether the additional spatial information that we have described here is in fact discriminatory, we investigated whether it improved our ability to specify true targets. Here we make a distinction between binding and regulation: binding does not necessarily imply regulation [10], [11], and indeed, possibly because it is typically informed by only a fraction of the promoter, our signature model is a relatively poor predictor of binding (figure S2). In contrast, we sought to determine whether our model improved the specificity of target prediction by identifying information that is preferentially found in regulatory targets. To this end, following the example of Westholm et al. [30], we measured the extent to which promoters matching these signatures exhibit expression changes when their target factor is deleted, comparing this aggregate change with the expression changes at promoters predicted as targets by spacing-agnostic models. We take these data from Hu et al., who used microarrays to measure genome wide expression changes in transcription factor deletion mutants [11].

To quantify the degree to which any given promoter matched a promoter signature, we calculated the expected value of the R “regulation” variable when the nucleotide variables are set equal to the sequence of the promoter. This gave us a metric by which to rank all promoters in the genome according to their match to the signature. We note that, presumably due to both the relatively small fraction of direct targets in the data and a considerable number of indirect targets exhibiting expression changes, neither ChIP nor computational methods predicted targets well as measured by a straightforward rank correlation with expression changes (table S1). However, by choosing an arbitrary cutoff point in this rank list, designating promoters above this cutoff as targets and those below as non-targets, we could compare the expression changes of targets favored by our model to those favored by other methods.

Using this framework we compared the specificity of our model against two other means of predicting factor targets, using the ChIP-chip data as a positive control. The first ranked promoters by the score of the highest-scoring single binding site they contained, and the second was a thermodynamic model which was able to take advantage of the information found in all of the possible sites to rank target promoters. Importantly, this model does not take into account site location and, unlike our model, handles site density only in an additive manner. For each computational method and ChIP-chip we arbitrarily chose to focus on its top fifty predictions (the results appeared robust to the choice of this cutoff; figure S3). We repeatedly sampled at random the same number of ORFs from the expression data to establish confidence intervals describing the null expectation that these predictions are not associated with expression. We disregarded factors that showed no relationship between ChIP-chip signal and expression; these factors might not be active in the condition tested (YPD), might not have a consistent role as an activator or repressor, or simply might not have sufficiently high quality binding data.

**Figure 5:**
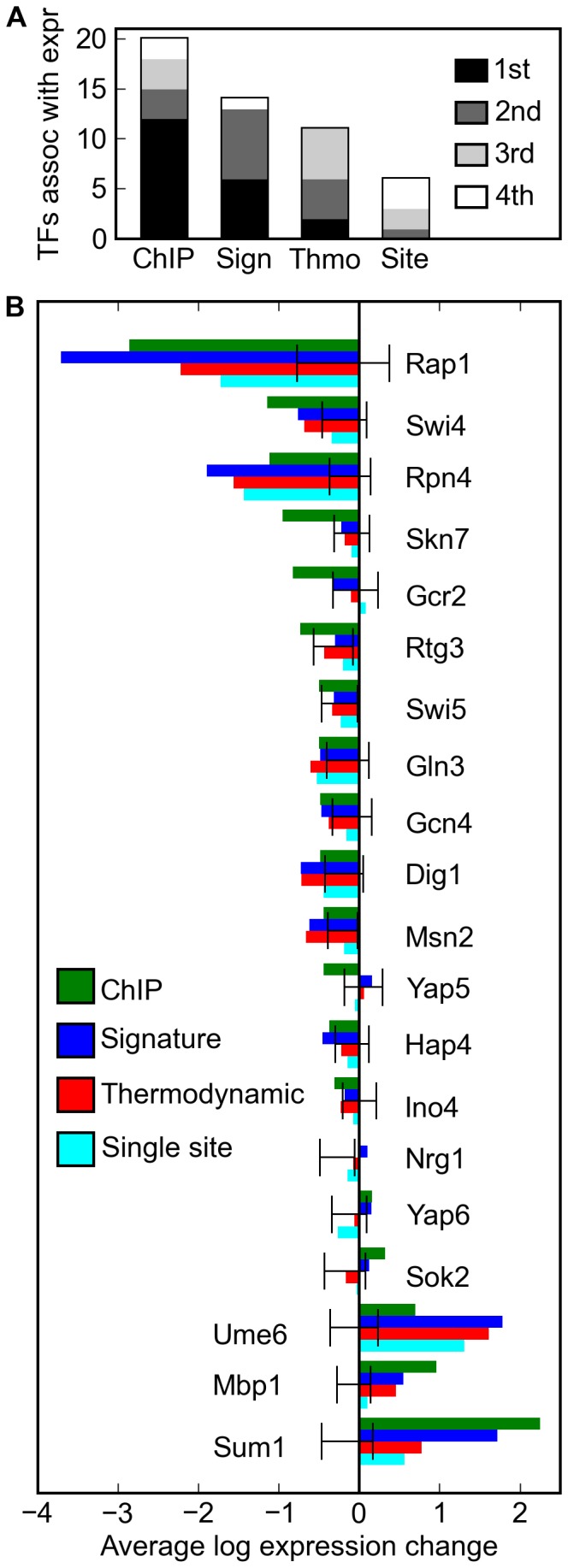
Spatial signature scores are correlated with expression change in transcription factor deletion mutants. (A) Considering the top 50 target promoters predicted by each of four methods, ChIP-chip, our sequence signatures model, a simple thermodynamic model, and a single site model, we plot the number of cases in which that method’s predicted targets for a transcription factor exhibited a significantly different average expression upon deletion of the transcription factor. We shaded each bar by the number of times that, for a given method and transcription factor, the magnitude of this average expression difference ranked 1st (black), 2nd (dark gray), 3rd (light gray), or 4th (white) among the methods significantly associated with expression for that transcription factor. The targets predicted by the spatial signature model typically showed a greater magnitude of expression change upon factor deletion than did the targets predicted by the thermodynamic model (p = .0176, see Methods), which in turn typically exhibited a greater magnitude of expression change than those targets predicted by the single site model (p = .0005). (B) For each transcription factor where the top 50 predictions of ChIP-chip were associated with expression, we plot for each method the average expression change of its top 50 predicted targets upon that factor’s deletion. We derived 95% confidence intervals by resampling 5,000 sets of 50 putative targets chosen at random from the expression dataset and calculating the average expression change in each of these. Note that if the measured changes in expression are widespread, as is the case for the Nrg1 deletion, it is possible for the confidence interval derived from this null distribution to not contain zero.

We found that the scores generated by our model were more likely to be significantly associated with expression than those generated by the other two computational methods, and that the ORFs ranked highly according to our model typically exhibited a larger expression change upon transcription factor deletion (fig. 5A): comparing the spatial signature model with the spatially-agnostic thermodynamic model, the signature model shows a greater magnitude of expression difference in twelve of the fifteen cases in which either model was significantly associated with expression (binomial p = .0176). Although our power to detect differences at the level of individual transcription factors is limited, the signature model showed a significantly greater association with expression than did the thermodynamic model in two cases (Rap1, p = .0012, and Sum1, p = .0027, see Methods). In an appreciable fraction of cases, the expression changes in the ORFs ranked highly by our model and sometimes other computational methods were larger than those found in ORFs appearing bound in ChIP-chip data, although this was not typical. As expected, the thermodynamic model consistently outperformed the model based only upon single sites (p = .0005). As a precaution against overfitting, we repeated this analysis with all ORFs used in training removed; these data supported the same conclusions (figure S4).

The performances of the computational models are clearly correlated (fig. 5B), and our model performed best relative to the ChIP data for transcription factors such as Rap1, Rpn4, and Ume6 where even single sites could predict targets well. We speculate that these more informative and presumably accurately described binding sites allow our model to more precisely describe their spatial distribution their spatial distribution in promoters. Conversely, and as we expect, the computational methods perform worst when the binding sites are not well described. Rtg3 and Gcr2 are the only two factors for which ChIP-chip but not any computational method, at any number of high-scoring ORFs tested, recovered a significant association with expression. Experiments conducted with protein-binding microarrays [38], [47] have cast doubt on the accuracy of the Rtg3 PWM we have used here, and while Gcr2 has not been investigated with these experiments, the PWM we use here is short and has been disputed by other computational work [48].

## Discussion

### Spatial Specificity Compensating for Poor Site Quality

Our principal finding is that well-specified promoter recognition signatures, often including restricted spacing, orientation bias, and, most importantly, multiple binding sites, are common and appear able to compensate for poor site specificity. As has already been discussed [6], the binding affinities of transcription factors in yeast, and in all eukaryotes, do not specify enough information to differentiate their targets from background DNA. It has been hypothesized [6], [23] that this handicap could be overcome through the use of multiple binding sites as a recognition signature.

By focusing on the characteristics of whole promoters, and not on the characteristics of individual binding sites, we are able to recover this property of binding site density, show it to be common, and demonstrate formally that it can indeed compensate for poor specificity of individual binding sites.

We did show for one factor, Msn4, that its spatial recognition signature as we have described it specifies almost exactly as much information as would be required to differentiate its true targets. This is an appealing result, and it echoes results for single binding sites in prokaryotes [7], but there are a number of reasons why we do not expect this to be a general property. First, there is no fast and accurate method for determining what a factor’s true target size is. The number of regions determined to be bound using ChIP-chip varies over more than an order of magnitude depending on the statistical and conservation criteria employed, and disrupting the target factor and searching for affected genes will inevitably recover a mixture of *cis*- and indirect *trans*-acting effects. Second, our model does not include properties of transcription factors already known in some cases to increase their specificity, such as association with different bound factors or tight spacing requirements between co-binding dimers. In an ideal model, we would have taken into account dependencies between positions of binding sites, although the relatively small magnitude of these dependencies [15]–[17], combined with the considerable computational complexity they would add to the model, led us to leave them out. Finally, proteins may dictate more specificity than they need to simply differentiate their targets. There is a relationship between information and affinity in individual binding sites [2]; it seems reasonable that this relationship might hold across promoters, with highly-specified promoters being bound a greater fraction of the time than weakly-specified ones.

### Consistency with Measurements of Synthetic Promoter Activity

Sharon et al. [49] recently published measurements of activity of thousands of short synthetic promoters that were designed to test the impact of some of the parameters we describe here (e.g. site orientation and density) on reporter gene expression as driven by a wide panel of transcription factors. Although they investigated some factors to greater depth than others, and many of those they investigated to the greatest depth were not described by our study, their work otherwise provides an ideal set of benchmarks for our model predictions.

Consistent with our results, they found a relationship between binding site number and expression that varies in magnitude from factor to factor: some transcription factors required relatively few binding sites before their cumulative effects reached a plateau, whereas other factors appeared to require many more. Cbf1, Gcn4, and Rap1, which we predicted to require few sites, fell into the former category, whereas Swi4, which we predicted to require more sites, fell into the latter. The remaining factor that was both described in our work and investigated in this manner in theirs was Fhl1, which had an unusual non-monotonic relationship between site number and expression change.

The authors also tested the impact of site orientation. As we do, they recovered a significant orientation effect for only a small fraction of their tested factors. Of that fraction, we also predicted the strand bias of Rap1 and Fhl1, although we did not predict the strand bias of Aft2, and their results were not consistent with our predictions of strand biases for Tec1 and Cin5.

### The Use of Binding vs. Coexpression Data

We used binding data as our source of training sets because it is most convenient, allowing us to train models for a large number of factors. It has a number of shortcomings. By focusing on the most strongly bound sequences in the genome, as we must when using these data, we may introduce a bias towards recovering strong sites or large numbers of sites. Perhaps more important, by choosing promoters for our training sets based only upon whether or not a factor is bound, we ignore the arguably significant role that spatial signatures may play in the determination of different expression patterns.

The context-specific properties of Rap1 are an illustrative example [18]. Rap1 binding sites are essential for the activation of many genes, including ribosomal protein genes and genes in the glycolytic pathway. It also is involved in gene silencing near telomeres and at the silent mating loci. Upstream of ribosomal genes, a particular pattern of binding, with sites arrayed in tandem on the coding strand, appears to be necessary for maximum expression. Upstream of glycolytic pathway genes, Rap1 usually has one binding site, without an orientation bias, located near one or more Gcr1 binding sites and is apparently essential for the binding of Gcr1. In telomeres, Rap1 appears to bind to a slightly different frequency matrix, perhaps brought about by changes in protein conformation. Several other proteins, such as Cbf1, share Rap1’s diversity of function and could potentially share its diversity of spatial signatures.

By focusing on binding instead of expression, we sum over all of these spatial signatures and likely reduce our ability to detect any of them. While we recover Rap1’s orientation bias upstream of ribosomal proteins, we mistakenly predict this feature to be general. Westholm et al. found a greater prevalence of orientation biases of transcription factor binding sites when they used coexpression rather than binding data [30], suggesting that promoter signatures may be more coherent in coexpression data sets. Although they are more limited, due to our inability to assign many factors to sets of coexpressed genes, the application and analysis of our model’s behavior on these sets is a natural next step.

### Use of Promoter Recognition Signatures as a Tool

We show that our model has generally higher specificity than a simple thermodynamic model in predicting transcription factor targets computationally and that, under certain conditions, it can refine the predictions of ChIP-chip. We would like to emphasize that our model as constructed is not an attempt to create a tool for transcription factor target prediction. As our goal was only to discover whether there exists spatial information that could help specify transcription factor binding sites, even if that spatial information is present in only a subset of targets, we paid no attention to sensitivity. Our model also currently requires a reference ChIP-based data set for training. While in principle the model could be trained on whole genomes without a reference ChIP training set, relying on the enrichment of the functional signature above the background, the background model used here is far too simple to represent the genome. However, the abundance of spatial information that we describe here shows that we can, in principle, increase the specificity of transcription factor target prediction by taking into account site context.

## Methods

### Preparation of Promoter Regions

We downloaded intergenic regions pre-screened for annotated features (‘NotFeature.fasta’) from the Saccharomyces Genome Database and used the results of [50] to remove the 5′ UTRs. In the small number of cases where data was unavailable, we removed the median 5′ UTR length from the beginning of the sequence. We trimmed these sequences to a maximum length of 1,003 base pairs, and we added masking 38% GC content sequence to the 5′ ends of sequences shorter than 1,003 base pairs. Finally, we discarded upstream regions that were noted in MacIsaac et al. [35] to be part of divergent promoters.

### Description of Model and Algorithm Implementation

The model is constructed as a directed graph closely related to a standard first-order Hidden Markov Model. There are four classes of variables. ‘R’ (‘regulation’) variables occur at the beginning of the HMM, take values 0 or 1, and determine whether at least one binding site will be emitted. These variables are included to account for the possibility of false positives in the training set. The ‘S’ (‘site’) variables form the backbone of the chain, each emitting one nucleotide ‘N’ variable. There are three values for the S variables that emit background sequence: b_0_ and b_1_ can transition to a frequency matrix state, whereas b_x_ can only transition to another b_x_ state. If the R variable is 0, the first S variable in the chain must take value b_x_, thereby preventing a binding site from being emitted. If the R variable is 1, then the first S variable can take a value of b_0_ or a frequency matrix state. The frequency matrix states (f_0_…f_w_, f_rc0_…f_rcw_) correspond to every possible position and reverse complement position in the frequency matrix. At the end of a set of S variables taking frequency matrix states, the next S variable must transition to either a b_1_ state or another frequency matrix state. b_1_ states do not transition back to b_0_ states, allowing the last S variable in the chain to specify whether or not at least one binding site was emitted. To ensure that promoters thought to be regulated by a factor, that is, taking a value of 1 for the R state, emit at least one binding site, we have incorporated a binary ‘C’ (‘consistency’) variable that takes value 1 if (a) if the final S variable takes the value b_1_ or corresponds to the end of a frequency matrix or (b) the final S variable takes value b_x_. We consider the C variable to have an observed value of 1, thus ensuring that the R state determines whether or not at least one site is emitted.

We also created a related ‘monosite’ model (as opposed to the ‘multisite’ model above) which emits precisely one binding site if the R state is 1. This is ensured by only permitting b_0_ states to transition to a series of frequency matrix states.

The nucleotides are emitted according to the frequencies in the given frequency matrix or from a background model weighted by GC content. In all above analysis, GC context was set at.38.

The value of the R state is given by:
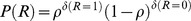



Frequency matrices can be emitted in either the forward or reverse orientation according to a parameter τ. The probability of emitting a frequency matrix from either b_0_, b_1_, or a finished series of frequency matrix states is:
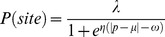



The value p corresponds to the position in the promoter, that is, the distance from the transcription start site. This value is multiplied by τ or 1-τ depending on the orientation of the matrix. This creates an approximately plateau-shaped distribution of binding sites, with μ and ω specifying the center and spread, respectively. The free parameter η either smooths or sharpens the boundaries of the plateau and was set to.1 for all described experiments.

We use the EM algorithm to fit the parameters, starting iterations from fifteen different sets of spatial parameters. The expectation is performed using message passing, and maximum likelihood estimates for ρ and τ are calculated analytically. We use simulated annealing to optimize λ, μ, and ω simultaneously. We implemented the algorithm in C using the GNU Scientific Library [51] and, for information and likelihood calculations, the GNU Multiple Precision Arithmetic Library [52]. The implementation is parallelized with MPICH [53] but can be run as a single process.

### Spacing Controls

Towards scrambling the spacing of binding sites as much as possible while leaving the strength and number of sites intact, we spatially scrambled the original training sets in an iterative fashion. First, we duplicated each set to a minimum size of 600 sequences. In each iteration, we picked a random subsequence from one of these sequences. The length of each such subsequence was randomly chosen to fall between arbitrary limits of 5 and 75. To ensure that moving this subsequence would not disrupt the binding signal, we checked if it had any binding sites of score zero or greater at its borders. If it did, we continued to pick random sequences until we found one having borders free of potential binding sites. We then chose another sequence at random of the same length using the same procedure. Once a matching sequence was found, we traded the two sequences. We repeated this process 100 million times. We fitted the same number of parameters to these models as were fitted to the originals, and we then repeated the optimization process on the original data while constraining the values of μ and ω to the shuffling-derived values. We determined significance using a likelihood ratio test with two degrees of freedom, determining for each factor whether the shuffling-derived values sufficiently described the distribution of binding sites present in the original data sets.

As described in the results section, we wished to exclude from our analysis factors that, perhaps due to a flaw in our background model, have a promoter signature appear enriched even in regions that are not bound. Unbound regions were defined as those which had a ChIP-chip binding p-value greater than.5 in every tested condition. For each factor, we assembled 20 sets at random from intergenic regions meeting this criteria, fitting each set starting from 20 different starting points. We used the value of ρ from each of these fittings to assess to what extent our model could detect a presumably false signal in each of these sets of presumably nonfunctional sequences. A factor’s signature was discarded if either: (a) finding the maximum trained ρ in each set, if the median of these maximums exceeded.15, or (b) any trained ρ value across these 400 fittings exceeded the ρ value found in the factor’s signature.

### Information Calculation

We used sampling to approximate the KL divergence between our promoter signatures and a simple background model specified only by GC content. The exact formulation of this divergence is specified as:
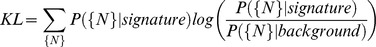



{N} here refers to the set of all nucleotide (‘N’) states in each promoter. Of course, integrating over all possibilities of {N} is impossible even for relatively short promoters. However, we can approximate this true value by sampling from the model, replacing P(mod) by 1/N (‘N’ here referring to the number of sampled promoters), below. {S} refers to a sampled promoter.
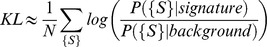



We verified the accuracy of this approximation by exactly calculating the information content of a short (ten base pair) model containing a single binding site for Ste12 and comparing it with values derived from sampling (data not shown).

### Recovery of Expression Change

We compared four methods in their ability to recover the expression changes found in transcription factor deletion mutants as described in Hu et al. [11]. Briefly, the authors used microarrays to survey 263 strains of yeast, each with a deletion of a single transcription factor, for expression changes relative to a strain with the transcription factor intact. For each method, we ranked all promoters according to the metrics described below.

For the matrix method, we ranked intergenic regions by their highest-scoring motif, for the ChIP-chip method, we ranked intergenic regions by the smallest p-value observed across conditions, and for our promoter signature method, we ranked intergenic regions by the expected value of the R state given by the model. While the ρ parameter does not affect rank, we calculated the expectations using ρ = .5.

The thermodynamic method relied on the framework described by Stormo [2]. Briefly, if we assume that each position in a binding site contributes independently to affinity, then we can describe the binding energy of a transcription factor and a binding site using the factor’s position weight matrix: the probability that the sequence is bound is proportional to the exponentiation of the score (below). We assumed for each factor that the cell contains a single protein competed for by all of the different intergenic regions. We ranked these regions by their probability of being bound by that factor. The probability of any given binding site being bound was set as:
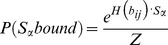
where Z is the sum of all the affinities found in the set and H in the weight matrix. Thus, the ranking metric, the probability that at least one binding site is bound, is given by:



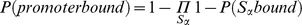



We used two different methods to compare the performance of the models. To compare the overall performance of two methods across all factors, we used a binomial test: we took as the sample size the number of factors for which either method recovered a significant association with expression, and we counted the number of times that one method’s predicted targets exhibited a larger average magnitude of expression difference.

We used bootstrapping to test whether the spatial signature method performed better than the thermodynamic method in predicting targets for each individual factor. We resampled the entire data set 10,000 times and retrieved the top 50 predictions of each method. To create a p-value, we counted the number of times in which the average magnitude of the expression change exhibited by these targets predicted by the thermodynamic model was greater than the magnitude of the change exhibited by targets predicted by the spatial signature model. Two factors showed a significant difference between the two methods (α = .05).

## Supporting Information

Figure S1 **The information of each spatial signature correlates with the information content of its component motifs.** For each transcription factor, we calculated the information content of its frequency matrix and the information content of its trained spatial signature model as depicted in figure 2. We calculated the information content of the frequency matrix according to Stormo [2], and we approximated the information content contained by the spatial signature model using a sampling procedure described in Methods. Note that the values are not comparable: the full model information describes a reduction of uncertainty across a whole 1 kb promoter region, while the motif information describes that reduction in a single binding site.
(TIF)

Figure S2 **Spatial signature models are relatively poor predictors of binding.** For each factor, we defined a ‘bound region’ as one having a binding p-value smaller than .05 in Harbison [34]. Then, ranking all promoters according to either their estimated binding probability in the thermodynamic model (red) or their expected value for the R variable in our spatial signature model (blue), we plotted a ROC curve. In most cases, the ROC AUC is substantially greater for the thermodynamic model’s predictions, although in some cases the signature model showed perceptibly higher sensitivity at the highest specificities (e.g. Skn7 and Sok2).
(TIF)

Figure S3 **Relative predictive ability of models robust to choice of rank list cutoff.** In figure 5, we showed the average expression change of the top 50 promoter targets as ranked by ChIP p-values (green), the expected value of the promoter’s R variable in the spatial signature model (blue), the binding probability as determined by a thermodynamic model (red), and the score of the top-scoring site in the promoter (cyan). Here we show results from the same analysis if the number of top-ranking promoters is designated as 10, 25, 50 (as shown in figure 5), 100, 200, or 400. The 95% confidence interval is shown in gray and calculated in the same manner as described in figure 5. The relative predictive ability of each method is in general robust to the choice of the rank cutoff.
(TIF)

Figure S4 **Exclusion of the training set does not affect perceived relative predictive ability of models.** We repeated the analysis of figure 5 in the main text, leaving out the promoters that had been used to train the spatial signature model. As they did in the original figure, the targets of the spatial signature model typically showed a greater magnitude of expression change upon factor deletion than did the targets predicted by the thermodynamic model (p = .0112, see Methods), which in turn typically exhibited a greater magnitude of expression change than those targets predicted by the single site model (p = .0352).
(TIF)

Table S1 Rank correlation of ChIP and computational model predictions with expression phenotypes. For each transcription factor in fig. 5, we computed the Spearman’s rank correlation between the scores assigned to each locus by an estimator of function (either ChIP, the spatial signation model (‘Sign’), or a thermodynamic model (‘Thmo’)) and the fold expression change measured at that locus upon that transcription factor’s deletion. These scores are the same as those discussed for figure 5 in the main text. For each test, we used all loci for which both a score and a measured expression phenotype were available. An asterisk marks values of the correlation coefficient significantly different from zero (p<.05, t test). All methods show a smaller number of significant associations with expression change as compared to the method outlined in the main text (11 vs. 20 for ChIP, 10 vs. 14 for the signature model, and 10 vs. 11 for the thermodynamic model), and these associations are less coherent: in two cases the sign of the significant correlation disagreed between the ChIP and a computational method (there were no such inconsistencies in the main text).
(DOCX)
