# Supplementary figures and images for: Spatial Promoter Recognition Signatures May Enhance Transcription Factor Specificity in Yeast
Source: PLoS One. 2013 Jan 8;8(1):e53778. doi: 10.1371/journal.pone.0053778 (PMC3540036; doi:10.1371/journal.pone.0053778)

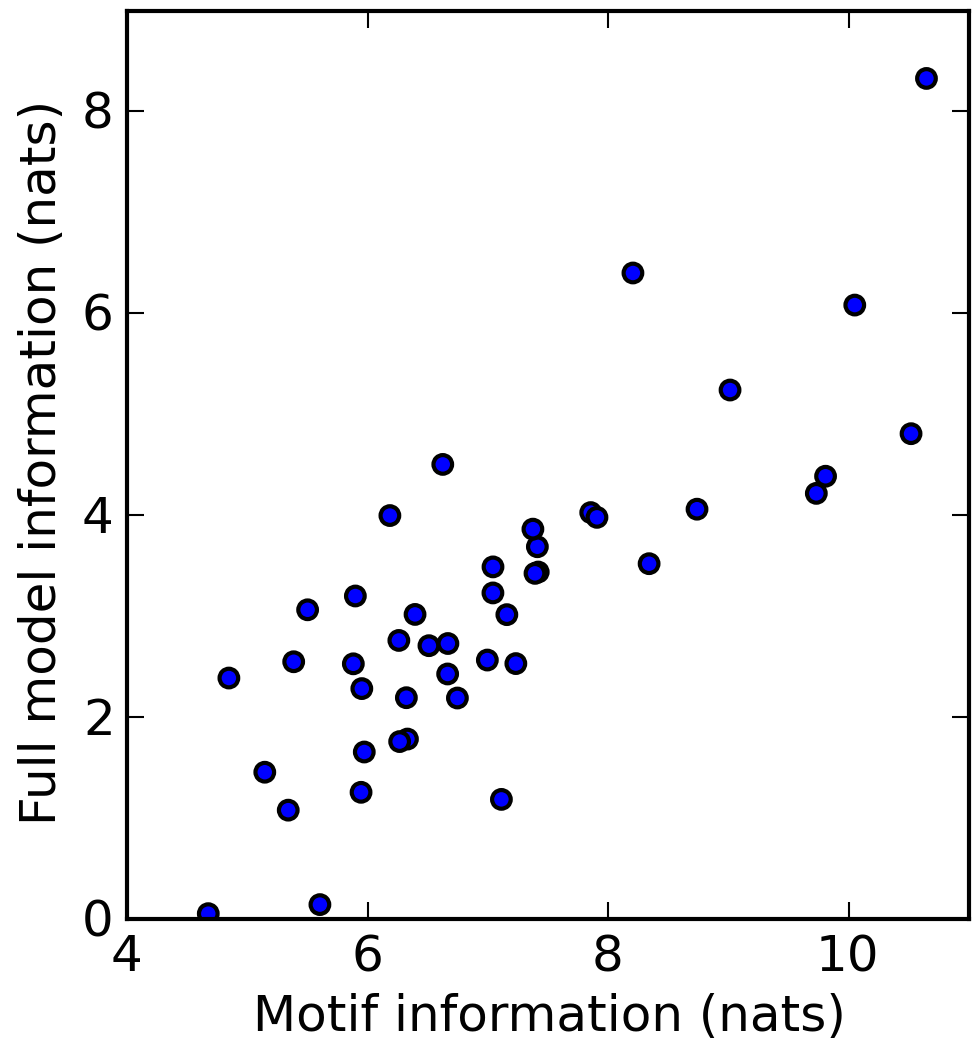

Supplement: Figure S1 — The information of each spatial signature correlates with the information content of its component motifs. For each transcription factor, we calculated the information content of its frequency matrix and the information content of its trained spatial signature model as depicted in figure 2. We calculated the information content of the frequency matrix according to Stormo [2], and we approximated the information content contained by the spatial signature model using a sampling procedure described in Methods. Note that the values are not comparable: the full model information describes a reduction of uncertainty across a whole 1 kb promoter region, while the motif information describes that reduction in a single binding site. (TIF) [file pone.0053778.s001.tif]

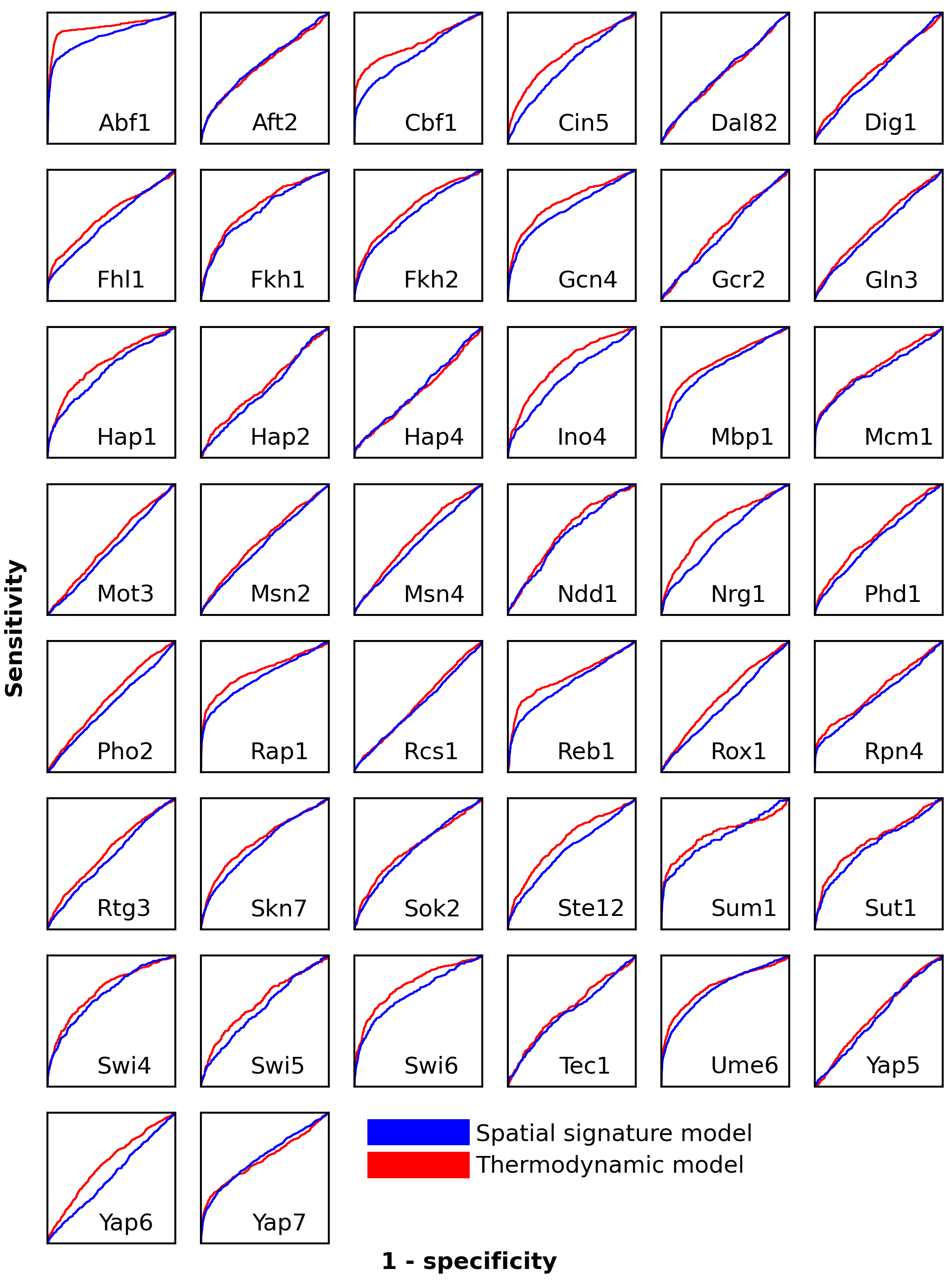

Supplement: Figure S2 — Spatial signature models are relatively poor predictors of binding. For each factor, we defined a ‘bound region’ as one having a binding p-value smaller than .05 in Harbison [34]. Then, ranking all promoters according to either their estimated binding probability in the thermodynamic model (red) or their expected value for the R variable in our spatial signature model (blue), we plotted a ROC curve. In most cases, the ROC AUC is substantially greater for the thermodynamic model’s predictions, although in some cases the signature model showed perceptibly higher sensitivity at the highest specificities (e.g. Skn7 and Sok2). (TIF) [file pone.0053778.s002.tif]

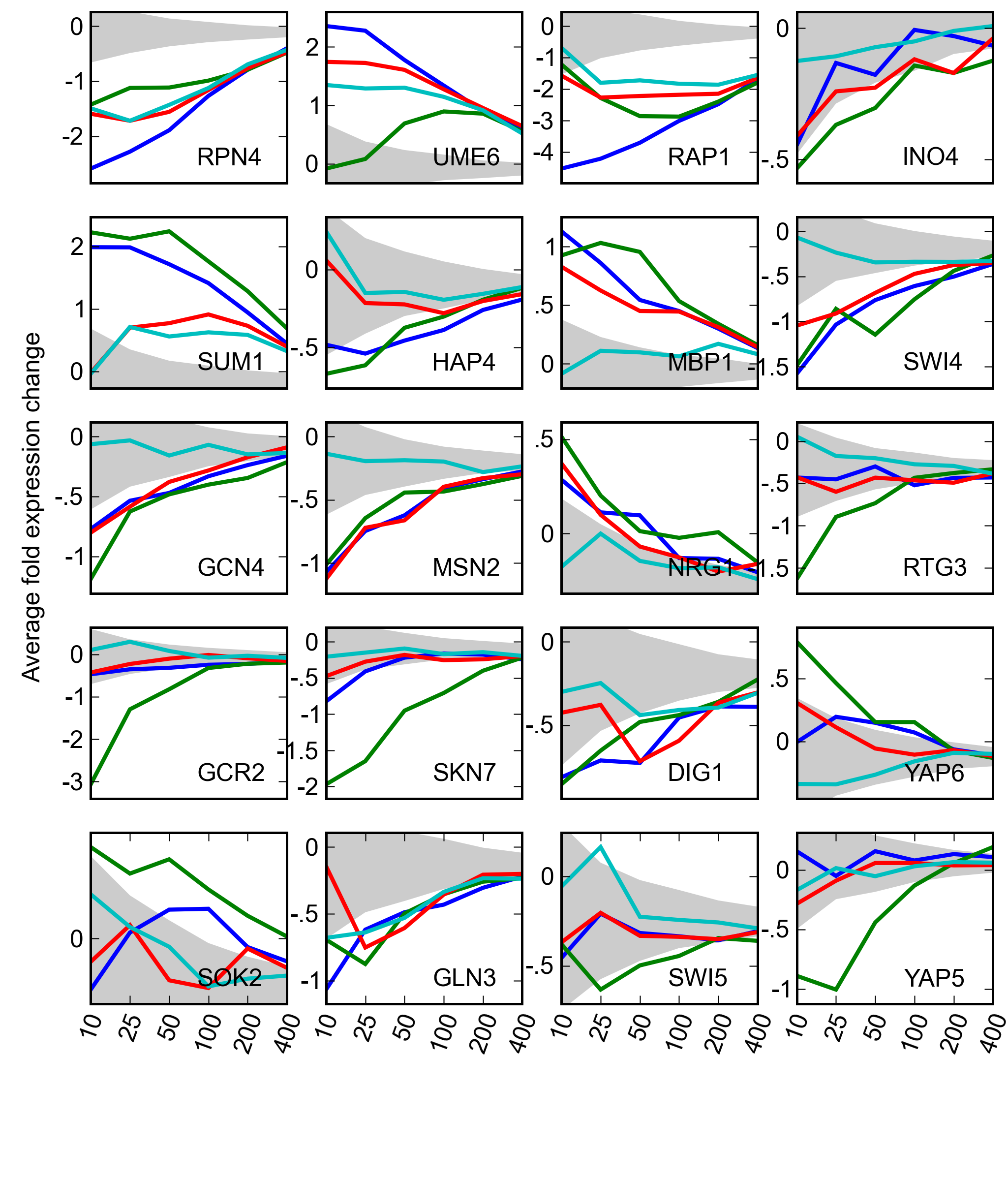

Supplement: Figure S3 — Relative predictive ability of models robust to choice of rank list cutoff. In figure 5, we showed the average expression change of the top 50 promoter targets as ranked by ChIP p-values (green), the expected value of the promoter’s R variable in the spatial signature model (blue), the binding probability as determined by a thermodynamic model (red), and the score of the top-scoring site in the promoter (cyan). Here we show results from the same analysis if the number of top-ranking promoters is designated as 10, 25, 50 (as shown in figure 5), 100, 200, or 400. The 95% confidence interval is shown in gray and calculated in the same manner as described in figure 5. The relative predictive ability of each method is in general robust to the choice of the rank cutoff. (TIF) [file pone.0053778.s003.tif]

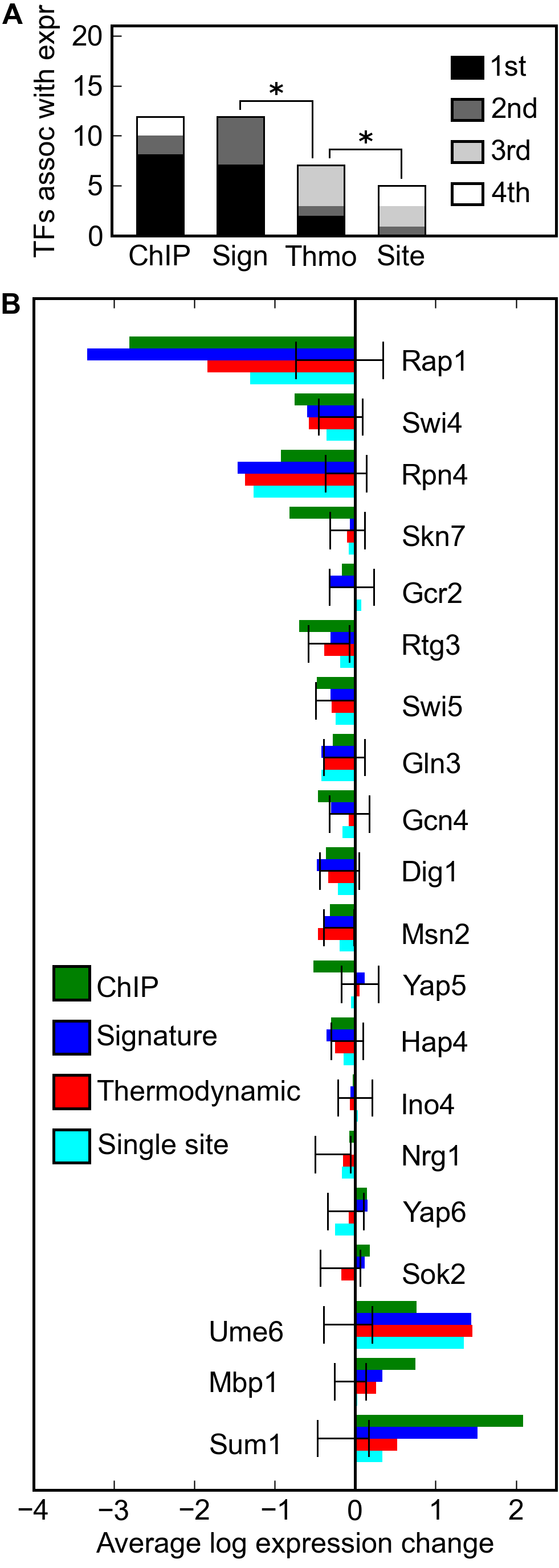

Supplement: Figure S4 — Exclusion of the training set does not affect perceived relative predictive ability of models. We repeated the analysis of figure 5 in the main text, leaving out the promoters that had been used to train the spatial signature model. As they did in the original figure, the targets of the spatial signature model typically showed a greater magnitude of expression change upon factor deletion than did the targets predicted by the thermodynamic model (p = .0112, see Methods), which in turn typically exhibited a greater magnitude of expression change than those targets predicted by the single site model (p = .0352). (TIF) [file pone.0053778.s004.tif]
