# Supplementary material for: Spatial Promoter Recognition Signatures May Enhance Transcription Factor Specificity in Yeast
Source: PLoS One. 2013 Jan 8;8(1):e53778. doi: 10.1371/journal.pone.0053778 (PMC3540036; doi:10.1371/journal.pone.0053778)
Supplement: Table S1 — Rank correlation of ChIP and computational model predictions with expression phenotypes. For each transcription factor in fig. 5, we computed the Spearman’s rank correlation between the scores assigned to each locus by an estimator of function (either ChIP, the spatial signation model (‘Sign’), or a thermodynamic model (‘Thmo’)) and the fold expression change measured at that locus upon that transcription factor’s deletion. These scores are the same as those discussed for figure 5 in the main text. For each test, we used all loci for which both a score and a measured expression phenotype were available. An asterisk marks values of the correlation coefficient significantly different from zero (p<.05, t test). All methods show a smaller number of significant associations with expression change as compared to the method outlined in the main text (11 vs. 20 for ChIP, 10 vs. 14 for the signature model, and 10 vs. 11 for the thermodynamic model), and these associations are less coherent: in two cases the sign of the significant correlation disagreed between the ChIP and a computational method (there were no such inconsistencies in the main text). (DOCX) [file pone.0053778.s005.docx]

**Supplementary table 1. Rank correlation of ChIP and computational model predictions with expression phenotypes.**

| Factor | ρ (ChIP) | N (ChIP) | ρ (Sign) | N (Sign) | ρ (Thmo) | N (Thmo) |
| --- | --- | --- | --- | --- | --- | --- |
| DIG1 | *0.0460 | 5944 | *-0.0289 | 5177 | 0.0001 | 5177 |
| GCN4 | -0.0157 | 5955 | *-0.0286 | 5182 | -0.0151 | 5182 |
| GCR2 | -0.0013 | 5960 | -0.0029 | 5188 | 0.0223 | 5188 |
| GLN3 | -0.0113 | 5939 | -0.0088 | 5168 | -0.0077 | 5168 |
| HAP4 | *0.0822 | 5846 | -0.0153 | 5086 | *-0.0435 | 5086 |
| INO4 | *-0.1016 | 5511 | *-0.0292 | 4873 | *-0.0545 | 4873 |
| MBP1 | 0.0154 | 5823 | *0.0508 | 5064 | *0.0408 | 5064 |
| MSN2 | 0.0021 | 5965 | *-0.0291 | 5189 | -0.0079 | 5189 |
| NRG1 | *0.0650 | 5895 | *0.0368 | 5137 | *0.0514 | 5137 |
| RAP1 | *-0.0711 | 5517 | *-0.0727 | 4814 | *-0.0820 | 4814 |
| RPN4 | *-0.0657 | 5615 | *-0.0292 | 4922 | *-0.0564 | 4922 |
| RTG3 | 0.0128 | 5911 | 0.0004 | 5143 | *0.0321 | 5143 |
| SKN7 | *-0.0416 | 5931 | -0.0229 | 5161 | 0.0027 | 5161 |
| SOK2 | *0.0308 | 5816 | 0.0153 | 5171 | *0.0303 | 5171 |
| SUM1 | *0.1079 | 5818 | *0.0503 | 5155 | *0.0337 | 5155 |
| SWI4 | -0.0239 | 5837 | -0.0055 | 5144 | 0.0183 | 5144 |
| SWI5 | -0.0047 | 5870 | -0.0189 | 5141 | 0.0096 | 5141 |
| UME6 | *0.1144 | 5932 | *0.1001 | 5167 | *0.1381 | 5167 |
| YAP5 | *0.0594 | 5915 | 0.0261 | 5156 | -0.0125 | 5156 |
| YAP6 | -0.0108 | 5728 | 0.0032 | 4981 | -0.0192 | 4981 |

For each transcription factor in fig. 5, we computed the Spearman’s rank correlation between the scores assigned to each locus by an estimator of function (either ChIP, the spatial signation model (‘Sign’), or a thermodynamic model (‘Thmo’)) and the fold expression change measured at that locus upon that transcription factor’s deletion. These scores are the same as those discussed for figure 5 in the main text. For each test, we used all loci for which both a score and a measured expression phenotype were available. An asterisk marks values of the correlation coefficient significantly different from zero (p < .05, t test). All methods show a smaller number of significant associations with expression change as compared to the method outlined in the main text (11 vs. 20 for ChIP, 10 vs. 14 for the signature model, and 10 vs. 11 for the thermodynamic model), and these associations are less coherent: in two cases the sign of the significant correlation disagreed between the ChIP and a computational method (there were no such inconsistencies in the main text).
